# 100 anastomoses: a two-year single-center experience with robotic-assisted micro- and supermicrosurgery for lymphatic reconstruction

**DOI:** 10.1007/s11701-024-01937-3

**Published:** 2024-04-06

**Authors:** Donata von Reibnitz, Andrea Weinzierl, Carlotta Barbon, Christian A. Gutschow, Pietro Giovanoli, Lisanne Grünherz, Nicole Lindenblatt

**Affiliations:** 1 Department of Plastic Surgery and Hand Surgery, University Hospital Zurich (USZ), Raemistrasse 100, 8091 Zurich, Switzerland; 2 Department of Visceral Surgery and Transplantation, University Hospital Zurich (USZ), Raemistrasse 100, 8091 Zurich, Switzerland; 3 University of Zurich (UZH), Raemistrasse 71, 8006 Zurich, Switzerland

**Keywords:** Supermicrosurgery, Robotic microsurgery, Robotic-assisted microsurgery, Lymphatic surgery, Robotic-assisted lymphatic surgery, Robotic-assisted supermicrosurgery

## Abstract

Robotic-assisted microsurgery has gained significant attention in recent years following the introduction of two dedicated microsurgical robotic systems specifically designed for this purpose. These feature higher degrees of movement and motion scaling which are useful tools, especially when performing surgery in areas of the body which are difficult to access. Robotic-assisted microsurgery has been implemented in lymphatic surgery as well as soft tissue reconstructive surgery at our institution over the past 2.5 years. Our study gives an insight into the details and outcomes of the first 100 consecutive (super-) microsurgical anastomoses in peripheral and central lymphatic reconstruction performed with the Symani^®^ Surgical System between 2021 and 2024. In total, 67 patients were treated, receiving robotic-assisted lymphatic reconstruction with lymphatic tissue transfer (LTT) and/or lymphovenous anastomoses (LVA)/lympholymphatic anastomoses (LLA). No anastomosis-associated complications were recorded postoperatively. The majority of patients reported a postoperative improvement of their lymphedema or central lymphatic disorder. In conclusion, we show the successful implementation of the Symani^®^ Surgical System into our clinical practice of lymphatic reconstruction. Although the necessary intraoperative setup and the use of intrinsic motion scaling lead to a slight increase in operating time, the presented study demonstrates the advantages of robotic assistance which becomes particularly evident in lymphatic surgery due to the involved deep surgical sites and the need for supermicrosurgical techniques.

**Supplementary Information:**

The online version contains supplementary material available at 10.1007/s11701-024-01937-3.

## Introduction

Robotic systems have been increasingly utilized in the field of plastic and reconstructive surgery in recent years to perform microvascular anastomosis for free flaps and lymphatic reconstructive surgery [1–4]. There are currently two CE-certified robotic systems that were developed specifically for microsurgery and supermicrosurgery. The MUSA robotic system (MicroSure, Eindhoven, Netherlands) has been implemented in 2020. Since its introduction, it has been successfully used in both preclinical and clinical studies with a focus on robotic-assisted lymphovenous anastomosis (LVA) [5–8]. The Symani^®^ Surgical System (Medical Microinstruments, Inc., Wilmington, USA) was introduced by us for reconstructive lymphatic surgery in 2021 [2]. Since then, its use has been expanded to almost all fields of reconstructive surgery including extremity reconstruction, autologous breast reconstruction, nerve reconstruction and microsurgical reconstruction of the central lymphatic system [9–13].

The Symani^®^ Surgical System offers advantages in terms of precision, dexterity and ergonomics for the microsurgeon. However, its high initial cost and ongoing expenses for consumables and maintenance pose challenges to its widespread adoption. Although we have observed a steep learning curve for experienced microsurgeons in terms of the time required per anastomosis, the overall operating time is increased by motion scaling as well as the preparation and handling of the robot by the surgical staff [9, 10]. Thus, a comprehensive benefit analysis is crucial in assessing the economic viability of integrating the Symani^®^ Surgical System into microsurgical practice. We have, therefore, conducted a thorough analysis of all patients who received lymphatic reconstructive procedures performed with the Symani^®^ Surgical System at our institution since the introduction of the system in 2021 in order to analyze the status quo and identify the current challenges.

## Patients and methods

We conducted a retrospective analysis of all patients in which microsurgical anastomoses in lymphatic reconstruction were performed with the use of the Symani^®^ Surgical System since its introduction at our institution in July of 2021. Approval for the study was granted by the Cantonal Ethics Committee of Zurich (04.01.2022 BASEC-Nr. 2021-02351). Written consent was obtained from all patients. Details regarding the robotic and technical setup can be found in our previous publication [2]. For the first cases, visualization was accomplished with the PENTERO^®^ 900 microscope (Carl Zeiss Meditec AG, Jena, Germany) or the VITOM 3D system (Karl Storz SE & Co. KG, Tuttlingen, Germany). In 2023, we transitioned to the KINEVO^®^ 900 optical microscope (Carl Zeiss AG, Oberkochen, Germany) which includes a 3D exoscope permitting the microsurgeon to control the robotic surgical system remotely. The senior author NL performed the majority of surgeries followed by LG. For all anastomoses, data on surgical technique, time as well as outcomes, e.g., complications, were recorded. For patients receiving lymphatic surgery, follow-up visits with volume measurements and photographic documentation of the affected limb were scheduled preoperatively and at standardized postoperative intervals. Subsequent visits were scheduled according to the clinical situation. Measurement analysis was performed in all patients for whom pre- and postoperative measurements (minimum 3 months following surgery) were available.

All data were analyzed using Microsoft^®^ Excel Version 2204 (Microsoft Corp., Redmond, WA, USA).

## Results

We analyzed 100 consecutive robotic-assisted micro- and supermicrosurgical anastomoses for lymphatic reconstruction performed between 2021 and 2024 at our institution. A total of 67 patients were treated, of those 50 were female and 17 male. The patients’ mean age was 48.3 years (range 8 months–88 years). The majority of patients received surgery for primary (*n* = 22) or secondary lymphedema (*n* = 33). In this cohort, the upper and lower extremities were affected in nine (13%) and 44 cases (66%) respectively, while two (3%) patients presented with isolated lymphedema of the genitalia. Other indications included central lymphatic reconstruction (*n* = 4), LVA following large volume soft tissue tumor resections (*n* = 6), as well as persistent seroma (*n* = 1) and lymph fistula (*n* = 1). With the exception of three LLA, all anastomoses were lymphovenous (*n* = 53) or arterial (*n* = 44) and the majority (*n* = 84) were performed end-to-end. Accordingly, 9-0 and 11-0 suture material was used most commonly. An overview of the information regarding anastomosis details can be found in Table 1.

**Table 1:** Patient demographics and anastomosis data (67 patients, anastomoses: *n* = 100)

	*n*	%
Mean age	48.3 years (range 10–88 years)	
Gender		
Female	50	75
Male	17	25
Indication for surgery		
Primary lymphedema	22	33
Secondary lymphedema	33	49
Central lymphatic duct anomalies	4	6
Lymphatic reconstruction during soft tissue tumor resection	6	9
Other	2	3
Mean surgery time	365.3 min (range 107–604 min)	
Type of anastomosis		
Lymphovenous (LVA)	53	53
Arterial	44	44
Lympholymphatic (LLA)	3	3
Anastomosis orientation		
End-to-end	84	84
End-to-side	16	16
Suture material		
8–0	4	4
9–0	29	29
10–0	17	17
11–0	48	48
Unknown	2	2

*LVA* lymphovenous anastomosis, *LLA* lympholymphatic anastomosis

Mean total surgery time was 365 min (range 107–604 min). This included flap harvest, preparation of the recipient site (s), indocyanine green (ICG)/patent blue lymphography, additional LVA (if patent vessels were found) and in some cases liposuction of the affected limb (s). Since 2020, we routinely perform lymphatic tissue harvest from the omentum majus in a laparoscopic manner in cooperation with visceral surgeons from our institution. The Symani^®^ Surgical System is draped and prepared intraoperatively as soon as the flap harvest is completed to keep any additional time needed for the robot setup at a minimum. The use of the KINEVO^®^ 900 optical microscope and 3D exoscope allow for the ergonomic positioning of the surgeon in front of a large display with remote use of the robot while a surgical assistant remains at the surgical site. In total, 23 patients received a combined approach lymphatic reconstruction including LTT and LVAs or LLAs. While 23 patients received only LTT, in 20 patients only LVAs/LLAs were performed. Lymphatic reconstruction was accompanied by liposuction of the affected limb (s) in 23 patients.

**Fig. 1:**
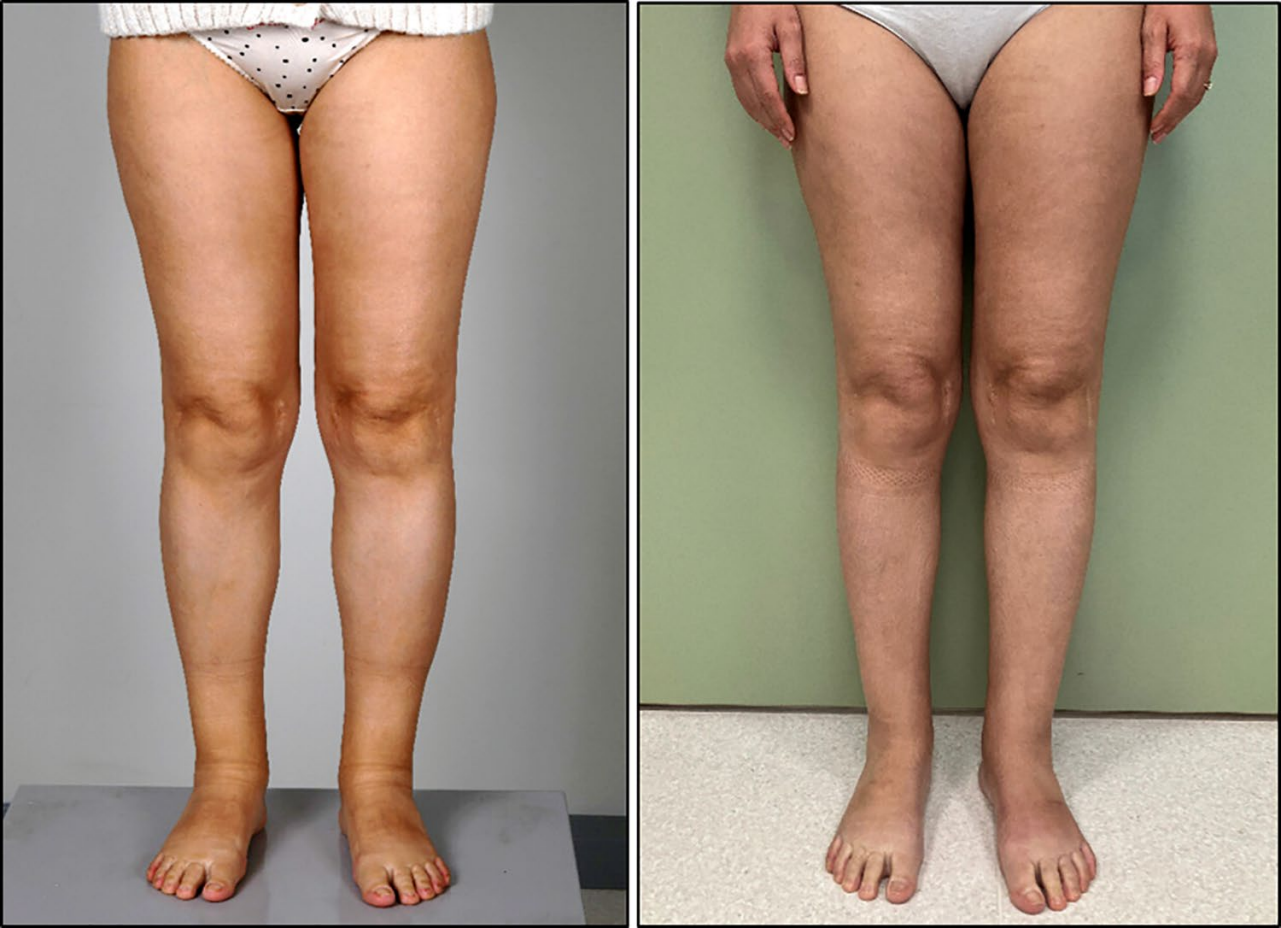
Clinical images of a patient receiving lymphatic tissue transfer (LTT) to both legs and an lymphovenous anastomoses (LVA) on both dorsal feet (**a**) preoperatively (**b**) 3 months postoperatively with a volume reduction of 1988 ml on the left and 785 ml on the right side

The mean follow-up time of the reported cases was 10.1 months (range 0–26 months). Postoperative measurements (minimum 3 months postop) were available for 39 limbs in total. Of seven affected arms, six showed a volume reduction (86%). The mean volume difference for all treated upper limbs, was − 281 ml (− 7.6% compared to initial limb volume). In the lower extremities, 23 of 32 legs showed a volume reduction (72%). A clinical example can be found in Fig. 1. The total mean volume difference per limb was − 288 ml (− 1.4%). Further details can be found in Table 2. In 33 patients for which data at 1 year postoperatively was available, 14 were able to reduce the compression garment class from the preoperative baseline.

**Table 2:** Postoperative limb volume measurements (*n* = 39 limbs)

	Mean volume difference in ml	Mean volume difference in % (volume difference/volume preoperative)	Range in ml		n (limbs)
			Min	Max	
Upper extremities	**− 281.98**	**− 7.60**	**121**	**− 1001.1**	**7**
LTT	− 239.33	− 4.87	− 49.5	− 520.5	3
LTT + LVA	− 303.30	− 8.97	121	− 1001.1	4
Lower extremities	**− 288.04**	**− 1.44**	**3995.1**	**− 4292.4**	**32**
LTT	− 970.77	− 7.21	310	− 2962.8	10
LTT + LVA	− 768.66	− 5.88	1949.3	− 4292.4	16
LVA	553.7	5.47	3995.1	− 1656.5	6

*LTT* lymphatic tissue transfer, *LVA* lymphovenous anastomosis, *LLA* lympholymphatic anastomosis

**Fig. 2:**
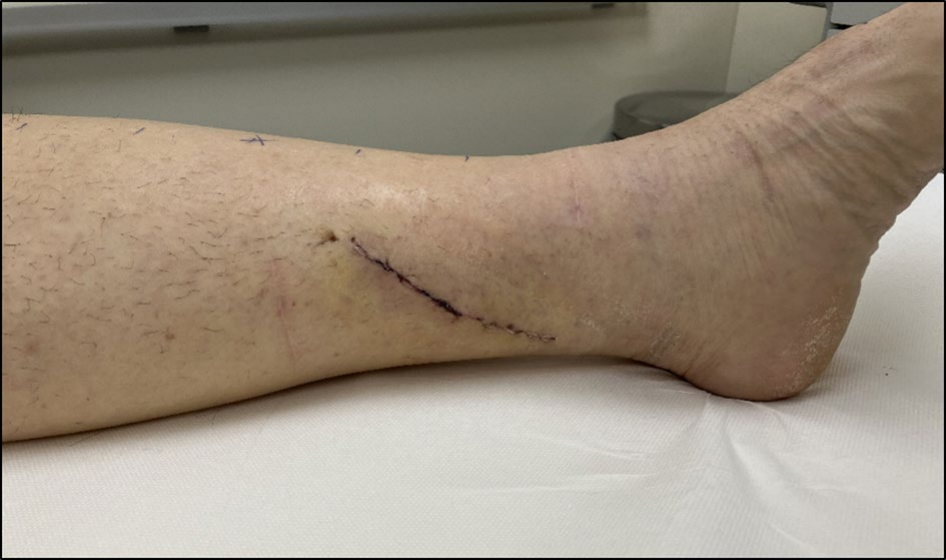
Surgical incision site for lymphatic tissue transfer (LTT) on the lower leg, two weeks postoperatively

Postoperative wound infections of surgical sites were documented in six patients (6.4% of all surgical sites). In two of the cases with registered infections, there were additional complications (wound dehiscence, development of a lymphocele) which required surgical revision of the wound. The remaining cases were treated with antibiotics alone. One patient developed a wound dehiscence without the need for surgical revision. Postoperative wound infection or delayed healing were almost exclusively recorded in patients receiving LTT to the ankle. Since then, the surgical protocol has been changed to transferring the omental flap to the middle to lower leg instead of the ankle area, which showed significantly improved wound healing [Fig. 2]. There was one postoperative hematoma requiring surgical evacuation. Two patients passed away during follow-up: one patient due to a nosocomial pneumonia and the other due to a metastasizing malignancy. None of the reported complications were associated with the use of the robotic system during the anastomosis or the laparoscopic harvesting of the lymphatic tissue.

## Case 1

**Fig. 3:**
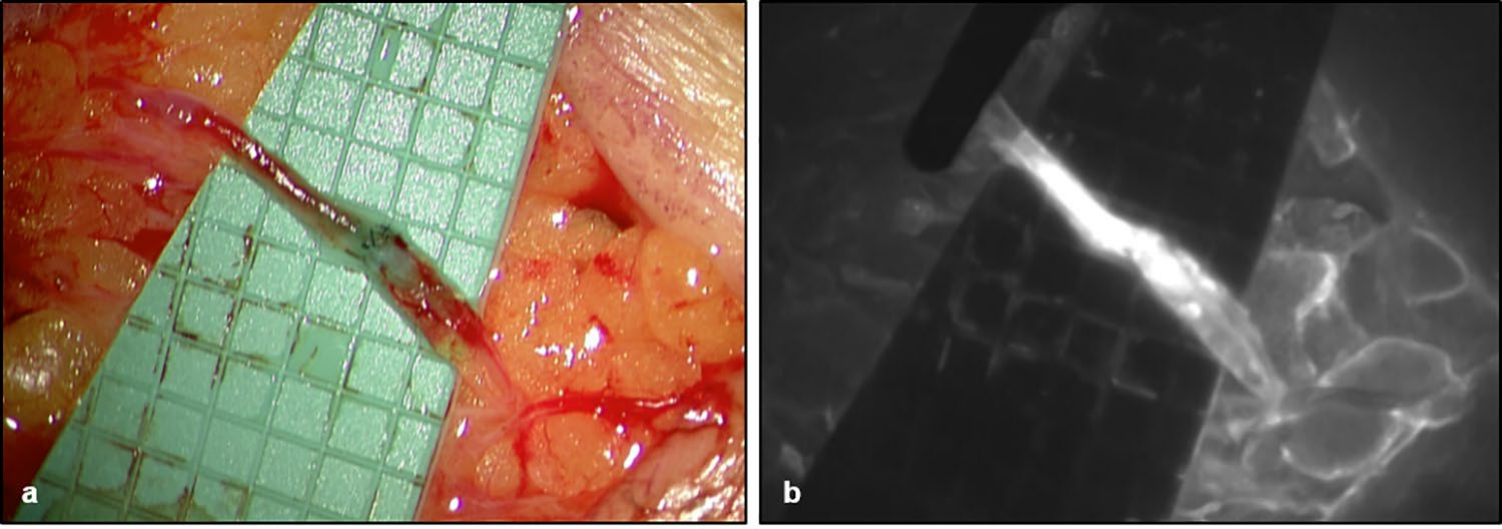
A patient receiving lymphatic tissue transfer (LTT) and lymphovenous anastomoses (LVA) to the left axilla and forearm, respectively (**a**) completed LVA (**b**) completed LVA after ICG injection

A 55-year old female patient presented with stage 2 secondary lymphedema of the left upper extremity. She had undergone mastectomy as well as a radical axillary lymphadenectomy on the left side followed by chemotherapy and radiation therapy due to breast cancer 25 years prior. The lymphedema had reoccurred 4 years prior following several episodes of erysipelas due to a superficial trauma. The dorsum of the hand as well as the forearm were mainly affected. At the time of her presentation, the patient had been wearing compression garments (class 2) fairly regularly, but was struggling with consistent use due to her occupation in healthcare. She was receiving manual lymphatic drainage once a month. No adequate control of symptoms (incl. pain, tension, and recurring erysipelas) could be achieved with conservative treatment. The patient underwent free vascularized lymph tissue transfer from the omentum majus to the left axilla. End-to-end arterial anastomosis of the right gastroepiploic artery to the thoracodorsal artery using Nylon 10–0 sutures was performed with the Symani^®^ Surgical System. A video of the completed anastomosis can be found in Supplementary Information 1. After patency of the anastomosis was confirmed, the patient also received lymphovenous anastomosis on the left distal forearm [Fig. 3a, b] and liposuction of the upper arm (300 ml lipoaspirate). There were no peri- or postoperative complications. The patient continued with compression treatment postoperatively and received an intensive course of manual lymph drainage and bandaging 1 month following surgery. 1 year postoperatively, the patient’s lymphedema has improved significantly. She has been able to stop wearing a compression garment and reduced the frequency of lymphatic drainage to once every other month. Volume measurements confirmed a reduction of 300 ml in comparison to the preoperative values without compression therapy.

## Case 2

**Fig. 4:**
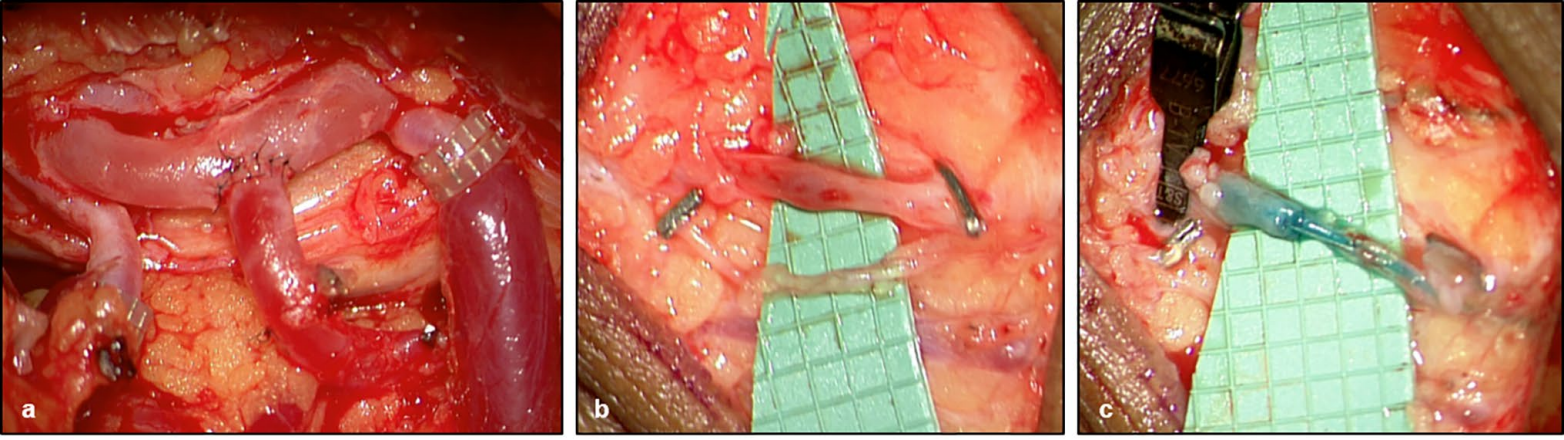
A patient receiving lymphatic tissue transfer (LTT) to both lower limbs and an lymphovenous anastomosis (LVA) on the right dorsal foot (**a**) completed arterial anastomosis and two venous coupler anastomoses on the left leg (**b**) preparation of lymph vessel and superficial vein for LVA (**c**) stenting of lymph vessel and vein prior to anastomosis with a polypropylene 5–0 suture

A 32-year-old female patient presented with a stage 2 lymphedema of the lower extremities as well as a stage 1 lipedema of the lower extremities. The patient reported that she had noticed a swelling of her legs since puberty, when she underwent an orthopedic procedure of both legs. Primary lymphedema had finally been diagnosed 1 year previously. She had since been wearing class 2 compression garments and received regular manual lymphatic drainage. Due to a progression of symptoms, the patient opted for a surgical treatment approach. The patient underwent free vascularized lymph tissue transfer from the omentum majus to both lower legs. End-to-side arterial anastomosis of the right gastroepiploic artery to the posterior tibial artery on the left leg was performed using the Symani® Surgical System and Nylon 9–0 sutures [Fig. 4a]. Patency of the anastomosis was confirmed. Additionally, a robotic-assisted lymphovenous anastomosis on the right dorsal foot was performed using 11–0 sutures [Fig. 4b, c]. To address the concomitant lipedema, liposuction of both thighs was performed with a total of 1100 ml lipoaspirate per leg. There were no peri- or postoperative complications. As per protocol, the patient continued with compressive therapy and received an intensive course of manual lymphatic drainage and bandaging 1 month after surgery. 6 months postoperatively the patient already showed a reduction of 500–700 ml volume per leg as well as a significant improvement of tissue density, despite being 5 months pregnant at that time. At the last follow-up, 1 year postoperatively, a total volume reduction of 2300 ml on the left side and 980 ml on the right side was registered. The patient is highly satisfied with the results, currently reports no residual symptoms of lymphedema, and has therefore been able to discontinue compression and lymphatic drainage.

## Discussion

The present study illustrates the successful implementation of the Symani^®^ Surgical System for microsurgical lymphatic reconstruction into clinical routines. Our data of the first 100 anastomoses suggest excellent surgical outcomes with high patient satisfaction. Robotic assistance in performing microsurgical anastomoses is associated with several advantages. Motion scaling helps reduce tremor and inefficient movement. Long instruments and a large range of motion including seven degrees of freedom improve precision especially while working in deep anatomical planes [10]. Remote operation with sterile manipulators enables the surgeon to work ergonomically while remaining sterile with a positive impact on endurance. Though drawbacks including an increased operating time, higher costs, difficulties in setup, and the necessity of additional training for surgical and technical staff have to be considered, the use of robotic systems has facilitated rapid advances in the field of lymphatic reconstructive surgery justifying its use.

Single cases and small case series of robotic-assisted free flap and lymphatic reconstruction using the Symani^®^ Surgical System have been published previously [2, 9–14]. At our institution, we have implemented the Symani^®^ in a variety of cases including lymphatic and soft tissue reconstruction. In our experience, the robotic system can be used in central as well as peripheral lymphatic reconstruction and is compatible with different microscopic and optical setups. Although the preparation of the Symani^®^ requires additional time and two surgical technical assistants/scrub nurses to drape the robotic system and the surgeons’ chair as well as the manipulators, we have observed that this time has decreased during the past 2 years. The work in an experienced team of assistants and nurses can prevent most setup issues and facilitate a successful handling of the robot. As more members of the microsurgical team have successfully trained to use the Symani^®^ Surgical System, the increased exposure has further helped to solidify clinical routines regarding the robotic setup. Certification for clinical use of the robotic system includes several sessions of intensive preclinical training using vessel models to simulate the intraoperative situation. We have previously published results showing a rapid learning curve for senior microsurgeons for the completion of robotic-assisted microsurgical anastomoses [9]. Similar results were reported in the first clinical trials using the MUSA system [7].

In our institution, lymphatic reconstruction is currently the predominant setting in which robotic-assisted microsurgery is performed, though indications keep expanding. These surgeries typically combine vascularized LTT and LVA, offering the possibility of using the robotic system for both arterial anastomoses, as well as LVAs. End-to-end and end-to-side anastomoses could be performed successfully using the robotic system. The different strengths of suture material (8–0 to 11–0) were used without complications. When using the needle-holder instrument with an integrated suture cut function, the presence of an additional surgical assistant at the surgical site is not necessary while performing vessel anastomosis. This makes it possible to simultaneously perform anastomoses on both extremities in a two team approach (one with the Symani^®^ system, the other with conventional manual microsurgery). Of note, the instruments of the Symani^®^ Surgical system also allow the use of even smaller suture material such as 12–0 nylon which has been recommended by other authors in order to improve accuracy of anastomosis in which vessel diameter is below 0.3 mm [15].

In our patient cohort, we showed mean volume differences of − 80 to − 1250 ml per limb compared to preoperative values, which corresponds to a relative difference of − 1 to − 10% of the treated limb to the preoperative measurements. As our cohort includes several patients with primary lymphedema in which both extremities are often affected, volume reduction calculations compared to a healthy contralateral limb were not feasible. This makes a literature comparison difficult, because most publications calculate volume reductions as a difference between the affected and the unaffected limb [16–21]. We registered wound healing complications in 6.4% of recipient or LVA sites in which the Symani^®^ was used, which compares favorably to postoperative complication rates of 7–17% after similar procedures without the use of robotic microsurgical assistance [16, 17, 19, 21]. Additionally, it has to be noted that many surgeons choose to close the recipient site with skin grafts instead of direct suture, as is the case in our institution [16, 19].

Our analysis has several limitations. Due to extensive changes in our surgical protocol when transitioning to the use of the Symani^®^ Surgical System, a comparison of robotic-assisted versus the current standard of manual anastomoses in the setting of lymphatic reconstruction at our institution is not feasible. When performing LTTs we routinely bury the flap without the possibility of flap monitoring, thus preventing an analysis of long-term flap survival in most lymphatic reconstruction cases.

To our knowledge, this study provides the largest case series of robotic-assisted microsurgical anastomoses in lymphatic reconstruction published so far. We could demonstrate the safety and efficacy of the Symani^®^ Surgical System in a spectrum of lymphatic reconstructive microsurgical procedures. Handling of the Symani^®^ requires practice for the surgeon as well as the surgical staff, but can be easily trained. Ergonomic positioning and intraoperative transition time improved over the course of the present case series. We saw no increase of anastomosis-related complications following microsurgical reconstruction when using the robotic system. Further exploration will be needed to better define and delineate surgical settings in which the robotic microsurgical systems provides explicit benefits over manual microsurgical anastomoses, but encouraging data continues to accumulate.

## Supplementary Information

Below is the link to the electronic supplementary material.

Supplementary file1 (DOCX 12 KB)

Supplementary file2 (MP4 12785 KB)

Supplementary file3 (MP4 143385 KB)

Supplementary file4 (MP4 143477 KB)

## Data Availability

No datasets were generated or analysed during the current study.
