# Supplementary material for: 100 anastomoses: a two-year single-center experience with robotic-assisted micro- and supermicrosurgery for lymphatic reconstruction
Source: J Robot Surg. 2024 Apr 6;18(1):164. doi: 10.1007/s11701-024-01937-3 (PMC10998780; doi:10.1007/s11701-024-01937-3)
Supplement: Supplementary file 1 — Supplementary file1 (DOCX 12 KB) [file 11701_2024_1937_MOESM1_ESM.docx]

Captions Supplementary Material

**Supplementary Information 1**

The video shows the completed arterial anastomosis and two venous coupler anastomoses after LTT to the left axilla

**Supplementary Information 2**

End-to-side arterial anastomosis of the right gastroepiploic artery to the posterior tibial artery on the left leg of the patient using the Symani® Surgical System and Nylon 9-0 sutures

**Supplementary Information 3**

Lympho-venous anastomosis of a 0.7 mm lymphatic vessel (right) to a 0.5 mm vein (left) using the Symani® Surgical System and Nylon 11-0 sutures. An intravascular stent (IVAS) is applied to stabilize the vessel for anastomosis
